# Molecular and immunophenotypic characterization of *SMARCB1* (INI1) - deficient intrathoracic Neoplasms

**DOI:** 10.1038/s41379-022-01133-4

**Published:** 2022-07-21

**Authors:** Martina Haberecker, Marco Matteo Bühler, Alicia Pliego Mendieta, Roman Guggenberger, Fabian Arnold, Eva Markert, Markus Rechsteiner, Martin Zoche, Christian Britschgi, Chantal Pauli

**Affiliations:** 1 Department of Pathology and Molecular Pathology, University Hospital Zurich, Zurich, Switzerland; 2 Department of Radiology, University Hospital Zurich, Zurich, Switzerland; 3 Institute of Pathology, Kantonsspital St. Gallen, St. Gallen, Switzerland; 4 Department of Medical Oncology and Hematology, University Hospital Zurich, Comprehensive Cancer Center Zurich, Zurich, Switzerland; 5 University Zurich, Zurich, Switzerland

**Keywords:** Translational research, Cancer genetics, Molecular biology

## Abstract

The switch/sucrose-non-fermenting (SWI/SNF) complex is an ATP-dependent chromatin remodeling complex that plays important roles in DNA repair, transcription and cell differentiation. This complex consists of multiple subunits and is of particular interest in thoracic malignancies due to frequent subunit alteration of *SMARCA4* (BRG1). Much less is known about *SMARCB1* (INI1) deficient intrathoracic neoplasms, which are rare, often misclassified and understudied. In a retrospective analysis of 1479 intrathoracic malignant neoplasms using immunohistochemistry for INI1 (*SMARCB1*) on tissue micro arrays (TMA) and a search through our hospital sarcoma database, we identified in total nine intrathoracic, INI1 deficient cases (*n* = 9). We characterized these cases further by additional immunohistochemistry, broad targeted genomic analysis, methylation profiling and correlated them with clinical and radiological data. This showed that genomic *SMARCB1* together with tumor suppressor alterations drive tumorigenesis in some of these cases, rather than epigenetic changes such as DNA methylation. A proper diagnostic classification, however, remains challenging. Intrathoracic tumors with loss or alteration of *SMARCB1* (INI1) are highly aggressive and remain often underdiagnosed due to their rarity, which leads to false diagnostic interpretations. A better understanding of these tumors and proper diagnosis is important for better patient care as clinical trials and more targeted therapeutic options are emerging.

## Introduction

The Switch/Sucrose-Non-Fermentable (SWI/SNF) complex, also known as BRG1/BRM- associated factor (BAF) complex, is involved in chromatin remodeling and transcriptional regulation, therefore contributing to cell differentiation and cell proliferation processes^1^. Over the past years, multiple SWI/SNF sub complexes and subunits have been discovered and their role in oncogenic processes described. The most relevant ones currently discussed and studied are *SMARCB1* (BAF47, INI1 or SNF5), *SMARCA4* (BRG1 or BAF190A), *SMARCA2* (BRM or BAF190B), *ARID1A* (BAF250A or SMARCF1) and *PBRM1* (BAF180). Nuclear INI1 (*SMARCB1*) is highly conserved and ubiquitously expressed in normal cells^2^. A morphological correlate associated with *SMARCB1* genomic alterations and immunohistochemical loss is the so-called ‘rhabdoid phenotype’, defined as the presence of eosinophilic cytoplasmic condensation adjacent to the nucleus. Loss of nuclear INI1 (*SMARCB1*) protein expression usually results from biallelic inactivation caused by different types of epigenetic or other deleterious genetic errors^3^. Complete loss of INI1 (*SMARCB1*) expression has been linked to a number of pediatric and adult mesenchymal tumors. The prototypical example is inactivation of *SMARCB1* (INI1) in pediatric malignant rhabdoid tumors (MRTs) and epithelioid sarcomas. *SMARCB1* deficient rhabdoid tumors are among the most aggressive and lethal pediatric cancers, however, mutations in *SMARCB1* also form the etiological basis of familial schwannomatosis, which is characterized by a predisposition to benign tumors^4^. Rare *SMARCB1* deficient tumors, more commonly occurring in adult patients, include synovial sarcomas, epithelioid malignant peripheral nerve sheath tumors, myoepithelial carcinomas, extraskeletal myxoid chondrosarcomas, chordomas, gastrointestinal stromal tumors (GIST) and ossifying fibromyxoid tumors.

Intrathoracic tumors associated with *SMARCB1* inactivation are exceedingly rare, but should be suspected when dealing with tumors arising in the soft tissue of the chest wall. The most frequent SWI/SNF complex subunit alteration in thorax, lung, pleural and mediastinal neoplasms is *SMARCA4*. *SMARCA4* - deficient undifferentiated tumors were recently recognized as a new entity in the WHO classification of thoracic tumors and are defined as malignant neoplasms with an undifferentiated or rhabdoid phenotype and deficiency of *SMARCA4* (BRG1)^5^. These tumors show molecular overlap with smoking-associated NSCLC harboring driver alterations in *STK11*, *KRAS*, and/or *KEAP1*^6,7^. Therefore, this entity is now termed *SMARCA4* deficient undifferentiated tumor rather than sarcoma and must be distinguished from *SMARCA4* deficient NSCLC, since *SMARCA4* mutations and/or loss of BRG1 expression occur in a subset of TTF1/p40 negative tumors, accounting for ~10% of poorly differentiated lung adenocarcinomas^8,9^. This type of carcinoma typically affects smoking men, and is associated with a short overall survival rate, regardless of disease stage^10^. Due to its aggressive behavior the identification of the inactivation and loss of BRG1 (*SMARCA4)* has become of interest for lung cancer management as recent preclinical studies have discussed therapeutic vulnerabilities that may overcome the inherently aggressive biology of SMARCA4 - deficient NSCLCs^11–13^.

*SMARCB1* deficiency has been mainly described in mesenchymal tumors, but next-generation sequencing studies have subsequently shown that *SMARCB1* alterations are also found in a subset of carcinomas, although at low frequency. They are often regarded as passenger events or second hits acquired at a later stage in tumorigenesis, as opposed to their initiating/driving role in MRT or epithelioid sarcoma. *SMARCB1* deficiency has been described in carcinomas of the gastro-entero-pancreatic tract, the head and neck region and in neoplasms of the genitourinary tract, representing a broad histomorphologic spectrum and polyphenotypic variations^14^. In short, alterations of SWI/SNF complex subunits, especially *SMARCB1* and *SMARCA4*, correlate commonly with distinct pathological features such as solid syncytial architecture, monotonous vesicular nuclei dotted with conspicuous nucleoli and/or rhabdoid cytoplasmic inclusions. Nonetheless, SWI/SNF complex subunit alterations are observed in both epithelial and mesenchymal tumors, both benign and malignant. Their recognition in routine pathology practice is challenging but possible, paving the way to targeted therapies^15^.

In this study, we molecularly and immunophenotypically characterized nine *SMARCB1* (INI1) deficient intrathoracic neoplasms and correlated this information with clinical presentation and outcome. We encountered four intrathoracic neoplasms with immunohistochemical INI1 (*SMARCB1)* deficiency in our diagnostic routine and consultation service. Each case has been identified as challenging from a diagnostic standpoint. Through a retrospective archival review using immunohistochemistry on TMAs and our in–house sarcoma database we found five additional cases.

## Materials and methods

### Patient cohort

Four intrathoracic neoplasms with INI1 (*SMARCB1)* deficiency were identified within our surgical and molecular pathology service including two consultation cases. Through a retrospective review of thoracic tumors in general from our institutional archive, additional five cases were identified. Three of these were found by immunohistochemical screening of thirteen TMAs by INI1 (*SMARB1*) immunohistochemistry, consisting of in total 1479 cases including pleural mesothelioma, non-small cell lung cancer (NSCLC), small cell lung cancer (SCLC), neuroendocrine tumors and large cell carcinoma (Supplementary Table 1). The remaining two cases were identified within our in-house sarcoma database. For all identified cases, the original diagnoses are included in Table 1. Morphologic features were assessed on hematoxylin and eosin-stained slides. Clinical information was obtained from the hospitals electronic medical records. All analyses were performed in our clinical laboratories. The study was approved by the local ethics committee (BASEC-2021-00417) and was conducted in accordance with local laws and regulations, including patients who signed our institution’s general informed consent.

**Table 1:** SMARCB1 deficient intrathoracic neoplasms: Patient information and clinical characteristics.

ID	Sex/Age (years)	Smoking pack-years	Follow-up/Month	Primary tumor location	Primary tumor size (cm)	Specimen	Lymph node metastasis	Site of distant metastasis	Primary diagnosis	Treatment
**1**	F / 20	0	DOD/16	Anterior mediastinum	~4^3^	Resection	None	OSS, PUL, OTH	Squamous cell carcinoma of the thymus	Adjuvant CT
**2**	M / 25	10	DOD/17	Pleura/thoracic wall/axilla	15.8^1^	Excisional bx	Mediastinal, hilar	LYM, PLE	Epithelioid sarcoma	Adjuvant CT
**3**	M / 51	35	DOD/6	Lung, central/ hilar	4–5^1^	Excisional bx	Lung station L2R, L4R	LYM, PLE	SWI/SNFT deficient Neoplasm	Adjuvant CT
**4**	M / 56	40	DOD/1	Pleura	Hemithorax^2^	Excisional bx	None	None	Mesothelioma	Adjuvant CT
**5**	M / 76	NA	NA/2	Lung, sub pleural	3.5	Lobectomy	None	NA	Malignant epithelioid tumor favoring epithelioid sarcoma	NA
**6**	M / 73	80	DOD/7	Lung, central	5	Lobectomy	Lung station L4R	LYM, HEP, ADR	Large cell carcinoma	Neo- and adjuvant CT
**7**	M / 64	13	DOD/10	Pleura	Hemithorax^2^	Excisional bx	None	None	Mesothelioma	Adjuvant CT
**8**	M / 72	30	DOD/53	Lung, central/ hilar	9	Lobectomy	None	PUL	LCNEC	Adjuvant CT
**9**	M / 77	0	DOD/4	Posterior mediastinum/esophagus	10^1^	Small biopsy	None	None	Epithelioid sarcoma, DDX: INI1 deficient esophageal carcinoma	Neoadjuvant CT

^1^radiological assessment, ^2^entire lateral parietal pleura, ^3^incomplete resection.

*AWD* Alive with disease, *bx* Biopsy, *CT* Chemotherapy treatment, *DDX* Differential diagnosis, *DOD* Died of disease, *F* Female, *M* Male, *NA* Not available, *ADR* Adrenal gland, *HEP* Liver, *LYM* Lymph node, OSS bone, *PLE* Pleura, *PUL* Lung, *OTH* Other (patient 1: soft tissue and nerve plexus), *LCNEC* Large cell neuroendocrine carcinoma.

### Immunohistochemistry

Immunohistochemistry was performed on 2 µm thick deparaffinized, rehydrated sections obtained from archived, paraffin-embedded blocks from each patient case using antibody-specific epitope retrieval techniques. Using an automated system for detection of the following primary antigens: INI1 (*SMARCB1*) (BD Biosciences, clone 25/BAF47, 1:300) and BRG1 (*SMARCA4*) (Abcam, clone EPNCIR111A, 1:50), pan cytokeratin (Dako, clone AE1/AE3,1:50), claudin4 (Invitrogen, clone 3E2C1, 1:200), BRM (*SMARCA2*) (Cell Signaling Technology, clone D9E8B, 1:800), TTF1 (Ventana-Roch, clone SP141, prediluted), CD34 (Ventana-Roche, clone QBEnd/10), Calretinin (Ventana-Roche, clone SP65, prediluted), Synaptophysin (Novacastra, clone 27G12, 1:50). For INI1, BRG1 and BRM, the reactivity was considered “deficient” if complete absence of nuclear staining in the background of intact positive controls (e.g. lymphocytes) was seen. Further immunohistochemical data was complemented or retrieved from the original pathology report. (Supplementary Table 2).

### Molecular analysis

#### Genomic profiling

8-μm thick unstained sections (5–10 slides each) of FFPE material were cut and macro-dissected according to corresponding hematoxylin and eosin slides to enrich specimens for tumor cells according to clinical protocols. Overnight proteinase K buffer digestion was followed by purification with the Maxwell^®^ RSC DNA FFPE Kit, PN: AS1450 kit. Double-stranded DNA was quantified by a Picogreen fluorescence assay using the provided lambda DNA standards (Invitrogen). 200 ng of dsDNA was fragmented to 50–1000 bp by sonication using the Covaris system prior purification using AMPure XP Beads (Beckman Coulter). Targeted Next-Generation Sequencing (NGS) was performed with the *FDA*-approved, broad comprehensive molecular diagnostic test FoundationOne®CDx assay (Foundation Medicine Inc., Cambridge, MA, USA). The assay sequences the complete exons of 324 cancer-related genes for the detection substitutions, insertion and deletion alterations (delins), and copy number alterations (CNAs) in 324 genes and select gene rearrangements, as well as genomic signatures including microsatellite instability (MSI), tumor mutational burden (TMB) and loss of heterozygosity score (LOH). Selected introns and promoter regions of 236 genes are also sequenced for the detection of gene rearrangements and fusions.

For the oncoprint generation, variants of unknown significance (VUS) were excluded and only significant variants and copy number alterations were included. Plots were generated using the *maftools* package in R. Molecular signatures were generated using the R package *MutationalPatterns* from the somatic gene variants obtained with high quality.

#### Methylation profiling

DNA was extracted from fresh frozen tumor sample (2/9) using the Promega Maxwell® RSC Tissue DNA Kit, PN: AS1610. If no fresh frozen tissue was available, FFPE material (see genomic profiling) was used utilizing the same DNA Extraction kit. 500 ng of genomic DNA from each sample was subjected to bisulfite conversion using an accredited in-house assay. The Infinium Human Methylation EPIC array was used to obtain genome wide DNA methylation profiles according to the manufacturer’s instructions (Illumina, USA). The quality of each sample was checked using the on-chip quality metrics and the R package *minfi* version 1.40^16^. IDAT files for all nine samples were uploaded to the DKFZ Sarcoma Classifier (version 12) (www.molecularsarcomapathology.org). All classifier results consisted of a suggested methylation class with an accompanying calibrated score. The calibrated score is a probability of the confidence for the given methylation class assignment. As defined by Koelsche *et al*, the classifier was only deemed to have made a successful prediction if the sample obtained a calibrated score of 0.9 or higher^17^. We further used the Epigenomic Digital Pathology (EpiDiP) platform (www.epidip.org) hosted from the Department of Pathology at the University Hospital Basel, Switzerland. All IDAT files were uploaded to EpiDiP. Conclusions made were based on UMAP results and copy number plots, also generated in EpiDiP.

## Results

### Patient cohort and clinical characteristics

**Fig. 1:**
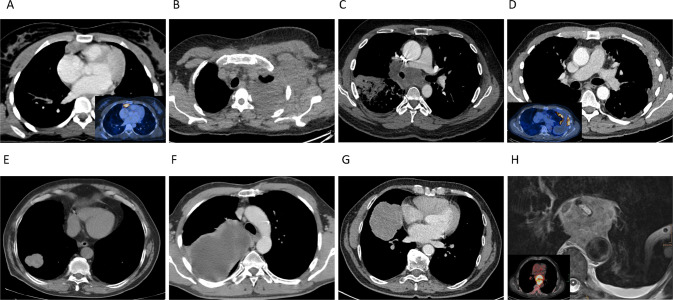
Representative computer tomography images of 8 patients, illustrating tumor localization and extension. **A** Patient 1: tumor localized in the anterior mediastinum (insert PET-CT scan). **B** Diffuse involvement of the thoracic wall with extension into the left axilla (patient 2). **C**, **E**, **G** Intrapulmonary localized tumor in patient 3, 6, 8, and 5 (not shown). Involvement of the pleura in patient 4 and 7. **D**, **F** Patient 9: tumor localized in the posterior mediastinum (insert PET-CT scan) (**H**).

In total, we identified nine intrathoracic neoplasms with immunohistochemical loss of INI1 (*SMARCB1*) (Supplementary Table 1), of which all were initially classified as intrathoracic neoplasms from either the lung (*N* = 3), lung/pleura (*N* = 1), pleura (*N* = 2), pleura/thoracic wall (*N* = 1) or mediastinum (*N* = 2). Patient information, clinical characteristics and original diagnoses are listed in Table 1. In our cohort, immunohistochemical INI1 (*SMARCB1*) deficient thoracic neoplasms occurred in one woman and eight men, ranging from 20 to 76 years of age (mean 57 years) at disease presentation. Smoking history was reported in six patients, ranging from 10 to 80 pack years (mean 34 pack years). For one patient smoking status was not available (patient 5) and 2 patients were documented as never-smokers. Based on computer tomography scans in eight patients, the tumors presented as lung (*N* = 3), lung/pleura (*N* = 1), pleura (*N* = 2), pleura/thoracic wall (*N* = 1) or mediastinal (*N* = 2) masses (Fig. 1). In one case (patient 2) the pleural mass further involved the thoracic wall and axilla, this patient presented with extensive repetitive pleural effusions positive for malignancy. The tumor sizes at presentation ranged from 3.5 to 15.8 cm (mean 7 cm). Imaging and clinical follow-up information were obtained for all nine patients, with a median follow-up of 14 months (range 1–53 months). All nine patients died of progressive disease, one patient (patient 5) was lost to follow-up due to an early transfer to another hospital (Table 1). Metastatic disease at presentation occurred in five patients, with four having lung or pleural involvement only. Two patients showed distant metastases in bone, soft tissue, liver and adrenal glands (patient 1 and 6). No brain metastases were documented in any of the nine patients. For therapy, the chemotherapy regimens varied, reflecting the original heterogeneous diagnoses of carcinoma, mesothelioma and sarcoma. The original diagnoses were mostly made in concordance with their particular anatomic site, all were, however, interpreted as high-grade malignancies (Table 1). Patient 1 initially presented in an outside hospital and was diagnosed with a thymic squamous cell carcinoma. The case was referred to us for molecular testing, which revealed a *SMARCB1* (INI1) homozygous loss that was accompanied by INI1 (*SMARCB1*) - loss in the complementary immunohistochemistry. Following re-evaluation by a soft tissue pathologist, the diagnosis of an epithelioid sarcoma, proximal type was concluded. The patient died of progressive disease 16 months after the initial presentation. Patient 4 died of localized disease within one month of initial presentation. Patient 9 was difficult to interpret as from a clinical presentation the tumor was located in the posterior mediastinum, growing almost circumferential around the esophagus. The patient passed away 4 months after initial presentation. Additionally, there were two cases (patient 4 and 7) with mainly in the pleural located neoplasms, none of which has a history of known asbestos exposition.

### Morphological features

**Fig. 2:**
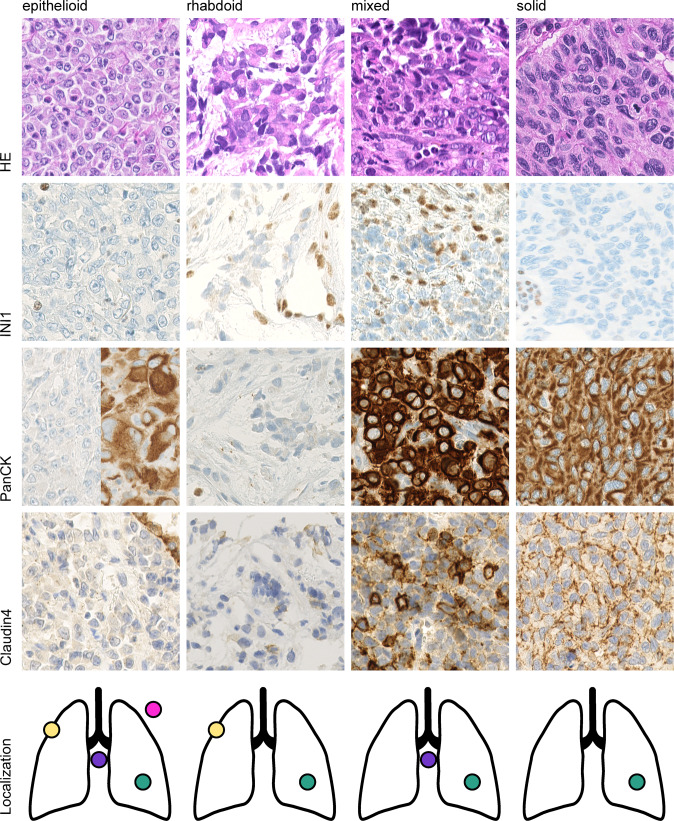
Histopathologic and immunhistochemical features of INI1 (*SMARCB1*) -deficient thoracic neoplasm. *Epithelioid* tumor cells with abundant eosinophilic cytoplasm and enlarged vesicular nuclei with prominent nucleoli (patient 1, 2, 5, 7). *Rhabdoid* morphology with hyaline cytoplasmic inclusions (patient 3, 4). *Mixed* morphology with discohesive- rhabdoid and discohesive epithelioid components (patient 6, 9). *Solid* tumor with cell-to-cell cohesion is evident in patient 8. Localization: pleural (yellow), intrapulmonary (green), mediastinal (purple), thoracic wall (pink). Magnification 400x.

Histologically, tumors harboring alterations in the SWI/SNF complexes usually show morphologic overlaps such as an epithelioid to rhabdoid morphology. Eight out of the nine tumors showed diffuse sheets of discohesive cells, of which two cases showed pure rhabdoid morphology with cells harboring distinctive hyaline cytoplasmic inclusions, and undifferentiated round to plasmacytoid cells with compressed crescent-shaped peripheral nuclei (patient 3 and 4). Four cases showed pure epithelioid morphology containing cells with abundant eosinophilic cytoplasm and enlarged vesicular nuclei with prominent nucleoli (patients 1, 2, 5, and 7). Overall, the tumor cells were relatively monotonous, with focally moderate pleomorphism seen in two cases, including scattered tumor giant cells. Two cases contained areas of mixed rhabdoid and epithelioid patterns (patient 6 and 9). Only one case (patient 8) showed a carcinoma-like solid growth pattern with cellular cohesion. Based on this we stratified the tumors in morphologic sub-groups: *epithelioid, rhabdoid, mixed and solid*. Patient 8 (solid morphology) suffered from an intrapulmonary mass containing large tumor cells with abundant cytoplasm and nuclear pleomorphism with variably coarse chromatin and positivity for synaptophysin, which is why it was initially classified as large cell neuroendocrine carcinoma (LCNEC) of the lung. Brisk mitotic activity and extensive necrosis were seen in all nine cases. None of the tumors demonstrated clear evidence of differentiation in the form of gland formation, keratinization or papillary structure. Representative hematoxylin and eosin images together with immunohistochemical stainings are shown for the morphologic sub-groups in Fig. 2.

### Immunohistochemical Features

The immunohistochemical findings are summarized in Table 2 and grouped according to the morphologic sub-group. As per inclusion criteria, all tumors showed complete loss of protein expression for INI1 (*SMARCB1*) with positive internal controls e.g. lymphocytes (Fig. 2). SWI/SNF complex subunit BRG1 (*SMARCA4*) was retained in all cases, whereas concomitant BRM (*SMARCA2*) loss was seen in four cases (patients 1, 4, 5 and 9) (Table 2). Pan-cytokeratin (AE1/AE3) expression was seen in half of the epithelioid (2/4) and all of the mixed (2/2) and solid (1/1) cases. In the majority of cases, strong and diffuse membranous pan-cytokeratin staining was seen, with some cases that demonstrated strong and diffuse cytoplasmic staining. Claudin4 was positive in the mixed (2/2) and solid (1/1) cases, concomitant with the pan-cytokeratin expression. The only case expressing TTF1, was an intrapulmonary tumor which has been negative for pan-cytokeratin (patient 5). One marker for neuroendocrine differentiation, synaptophysin, was positive in two cases (patient 5 and 8), in a pan-cytokeratin negative tumor with epithelioid morphology and a pan-cytokeratin negative tumor with solid morphology. Another two cases, both with a predominant pleural tumor mass (patient 4 and 7), were initially classified as mesothelioma, although all mesothelial markers such as calretinin, WT1, D2-40 and CK5/6 tested negative and BAP-1 and MTAP were retained (Table 2 and Supplementary Table 3). Based on methylation array data, which revealed closely related tumor methylation classes, further immunohistochemical markers were performed for individual cases and are listed for review in Supplementary Table 3.

**Table 2:** SMARCB1 deficient intrathoracic neoplasms: Immunhistochemical findings.

Group ID	epithelioid				rhabdoid		mixed		solid
Patient ID	1	2	5	7	3	4	9	6	8
**SMARCB1 / INI1**	-	-	-	-	-	-	-	-	-
**Pan-CK AE1/AE3**	+	-	-	+	-	-	+	+	+
**Claudin4**	-	-	-	-	-	-	+/-*	+	+
**SMARCA4 / BRG1**	+	+	+	+	+	+	+	+	+
**SMARCA2 / BRM**	-	+	-	+	+	-	-	+	+
**TTF1**	-	-	+	-	-	-	-	-	-
**CD34**	-	-	+	-	-	-	-	-	-
**Calretinin**	-	-	-	-	-	-	-	-	-
**Synaptophysin**	-	-	+	-	-	-	-	-	+

- = loss of expression/ negative staining, + = retained expression/ positive staining, +/- *= positive in one-half of the tumor cells.

### Molecular analysis

**Fig. 3:**
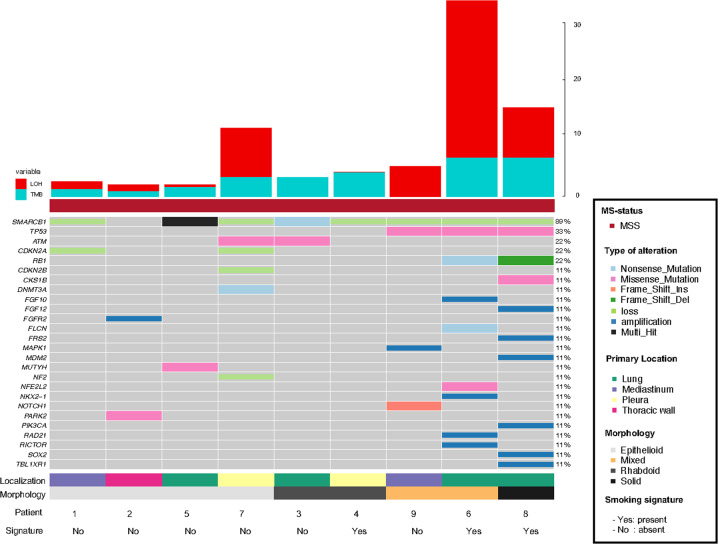
Oncoprint showing the distribution of genomic alterations found in the cohort. Annotations include following biomarkers; Tumor mutational burden (TMB) loss of heterozygosity (LOH), mismatch repair status (MS). Additionally tumor localization, smoking signature status and morphology subgroup are included.

**Fig. 4:**
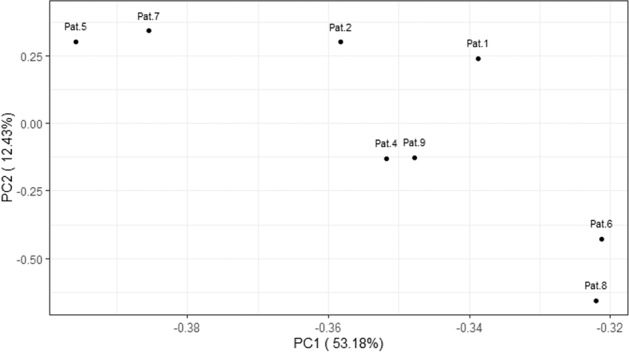
Principle component analysis (PCA). The PCS of 8 samples shows clustering of patients and separates patient 6 and 8 from the rest of the group. Patient 3 is not listed due to QC fail.

Genomic panel testing in all nine cases was performed using the FoundationOne®CDx Assays (Fig. 3). Homozygous *SMARCB1* loss was observed in six cases and *SMARCB1* mutations in two tumors (patient 3 and 5). In one case, patient 2, we detected within the target range from chr22:24176586 to chr22:24176715 on hg19 in *SMARCB1* (NM_003073), a shift below the median copy number range. However, as this target is relatively close to CN = 1, it did not meet the full criteria of a homozygous loss computationally. The target comprises a region downstream of exon 9 in the *SMARCB1* gene and appears to be entirely comprised of the untranslated gene region. It is well conceivable that this single target represents a single copy loss of one allele of *SMARCB1* thus leading to loss of immunohistochemical protein expression. All tumors were microsatellite stable and showed a tumor mutational burden <7 mut/Mb. In eight tumors, we detected a loss of heterozygosity (LOH) score below 1.5%, while one tumor presented a LOH score of 26.5% (patient 6). All detailed results, including the variants of unknown significance (VUS) are shown in Supplementary Table 4. Genomic signature analysis revealed absence of smoking signature in six patients (smokers and non-smokers) and interestingly only low contribution in 3 patients with known smoking history and more than 30 pack-years (patient 4, 6, and 8, Supplementary Fig. 1). In none of the patients we found mutations such as in *KRAS, KEAP1* and *STK11* that are usually present and typical of smoking-related NSCLC. Also, no other common lung adenocarcinoma driver mutations such as *EGFR* or *ALK* were detected. All nine samples were subsequently submitted for DNA methylation profiling using the Infinium Human Methylation EPIC Bead Chip array for analysis applying the sarcoma classifier and copy number analysis (Table 3 and Fig. 4). We excluded one sample (Patient 3) from further analysis due to poor DNA quality and CNV plots of three additional patient (patient 1, 6 and 8) were also deemed not evaluable due to low quality. We attempted a tumor classification by using the DKFZ Sarcoma Classifier platform version 12 (www.molecularsarcomapathology.org) and the EpiDiP server (www.epidip.org) hosted from the University Hospital Basel, Switzerland. Using the DKFZ sarcoma classifier no successful prediction (calibrated score >0.9 score) could be established for any of the nine cases. This calibrated score indicates the probability of the confidence for the given methylation class assignment. Our cases had a median score of 0.6 (0.4–0.89) excluding two samples that failed to provide a score. However, seven of the nine tumors were classified as closely related tumor methylation classes such as for example epithelioid sarcoma. The calibration scores and related methylation classes are provided in Table 3. Copy number profiles are provided in Supplementary Fig. 2. None of the cases showed methylated CpGs in the putative promotor region of the *SMARCB1* gene, in line with earlier reports^18^. In patient 4, the only detected and driving alteration was a homozygous loss of *SMARCB1* (INI1) what is reflected in the related methylation class of a malignant rhabdoid tumor. This patient’s tumor showed a slight smoking signature but has no smoking related alterations detected otherwise. In five other patients (patients 1, 2, 3, 5, 7) *SMARCB1* (INI1) alterations (loss and mutations) are accompanied by the loss or inactivation of genes with a tumor suppressive role such as *CDKN2A*, *ATM, NF2* and *PARK2* representing a complex genotypes such as for example seen in epithelioid sarcoma^19^. These tumors were wild type for the *TP53* gen as more commonly described in epithelioid sarcoma. Methylation analysis of these 5 tumors showed an enriched relation to known *SMARCB1* (INI1) driven entities. In patients 6, 8 and 9 we had concomitant *SMARCB1* (INI1) loss and a *TP53* missense mutation together with the inactivation of other tumor suppressors such as *CDKN2A* and *RB1*. Interestingly, patient 9 related closest to the group of epithelioid sarcoma despite harboring a *TP53* mutation. A principal component analysis (PCA) showed clustering of some patients (patient 4 + 9, 5 + 7 and 6 + 8). Interestingly, patient 6 and 8 seem to cluster separately from the rest of the cohort (Fig. 4). These two patients harbor the highest mutational load with alterations in *TP53* and *RB1* and show a smoking related signature.

**Table 3:** Methylation profiling of SMARCB1 deficient intrathoracic neoplasm.

Group ID	Patient ID	TC (%)	Methy. Quality (*p*-value)	*DKFZ sarcoma classifier*			*EpiDiP classifier*	Initial diagnosis
				Calibrated score	Related Methylation class	Interpretation	Related Methylation class	
**epithelioid**	1	35	0.0192	0.52	Epithelioid sarcoma	No match	No match	Squamous cell carcinoma of the thymus
**epithelioid**	2	45	0.0032	0.4	Langerhans cell histiocytosis	No match	No match	Epithelioid sarcoma
**epithelioid**	5	50	0.0001	0.89	Chrodoma (dedifferentiated)	No match	No match	Malignant epithelioid tumor favoring ES
**epithelioid**	7	60	0.0003	0.6	Epithelioid sarcoma	No match	No match	Mesothelioma
**rhabdoid**	3	80	>0.1*	none	Not to classify	No match	No match	SWI/SNFT deficient Neoplasm
**rhabdoid**	4	85	0.0012	0.74	Malignant rhabdoid tumor	No match	No match	Mesothelioma
**mixed**	9	80	0.0024	0.86	Epithelioid sarcoma	No match	No match	ES, DDX: INI1 deficient esophageal carcinoma
**mixed**	6	60	0.0253	none	Not to classify	No match	No match	Large cell carcinoma
**solid**	8	60	0.0202	0.47	Family plexus tumor	No match	No match	LCNEC

*Failed quality score.

*DDX* Differential diagnosis, *ES* Epithelioid sarcoma, *LCNEC* Large cell neuroendocrine carcinoma, *TC* Tumor content.

## Discussion

The discovery that genes encoding subunits of SWI/SNF complexes show genomic alterations across a wide variety of cancer types is about a decade old and consequently, our understanding of the mechanisms and the potential therapeutic implications remains in its infancy^20^. SWI/SNF complex-deficient carcinomas and mesenchymal tumors commonly share a discohesive epithelioid or rhabdoid morphology and this should guide the use of markers such as INI1 (*SMARCB1*) and BRG1 (*SMARCA4)* in the diagnostic work-up. For thoracic epithelioid neoplasms with lack of TTF1 positivity, a BRG1 (*SMARCA4*) staining should be considered in order to elucidate a TTF1 negative, *SMARCA4* - deficient non-small cell lung cancer^21^. They usually lack undifferentiated/sarcomatoid features. In tumors with undifferentiated components such as round cell or rhabdoid morphology and loss of BRG1 (*SMARCA4*), the diagnosis of a *SMARCA4* - deficient undifferentiated tumor should be made. *SMARCA4* - deficient undifferentiated tumors in the thorax are usually smoking related^9^. The investigation of an INI1 (*SMARCB1*) deficient neoplasm is highly recommended in such cases when BRG1 (*SMARCA4*) is retained. This should be considered independent of the age of the patient and not be misguided by the location and the cytokeratin status of the tumor.

We here presented nine patients with an intrathoracic neoplasm with immunohistochemical loss of INI1 (*SMARCB1*). We investigated if these intrathoracic *SMARCB1* - deficient neoplasms represent an own unique entity. All of our cases showed retained expression of BRG1 and no genomic alteration in *SMARCA4* or methylation events, clearly demarcating them from the spectrum of *SMARCA4* - deficient non-small cell lung cancers and *SMARCA4* - deficient undifferentiated tumors^9^.

Eight out of nine cases show overlapping morphology with both categories, SWI/SNF complex deficient carcinomas and sarcomas. The differentiation between these two categories is particularly challenging. We therefore evaluated Claudin-4, a useful marker in the distinction between carcinoma and mesothelioma^22^, but also between carcinoma and sarcoma^23^. In a study by Schaefer et al. Claudin-4 expression was detected in 80% of SWI/SNF complex-deficient undifferentiated carcinomas compared with only 4% of sarcomas with epithelioid morphology. However, carcinomas with complete loss of Claudin-4 expression have also been described. In our cohort, Claudin-4 and pan-cytokeratin AE1/AE3 co-expression was identified in three tumors with mixed epithelioid/rhabdoid or solid growth pattern. In two of them (patient 6 and 8), the strong Claudin-4 staining was in favor of a carcinoma diagnosis. In our opinion Claudin-4 is helpful in the differentiation of *SMARCB1* (INI1) - deficient carcinoma and sarcoma.

Furthermore, we explored genomic and methylation profiling and believe that a proper molecular work up can contribute to a more accurate classification. Nevertheless, using methylation profiling was not straightforward. First, some of our samples had a low tumor purity with probable negative impact on the calibration scores. However, recent data suggests that this should not affect the accuracy of the prediction^24^. Second, although the EpiDip classifier includes a broad tumor entity spectrum accounting for many carcinoma subtypes, no definite match was found here (Table 3).The DKFZ classifier does not yet include all tumor entities and tumor subtypes, why predictions above the threshold are not to be expected for all tumors in this classifier. Although all nine cases failed to be successfully classified (calibrating scores: < 0.9), it is noteworthy that seven cases were matching closely to related tumor entities. In the majority of cases we see a relation to known *SMARCB1* driven sarcomas such as MRT (patient 4) and epithelioid sarcoma (patients 1, 2, 3, 5, and 7). MRT of the mediastinum is a rare aggressive tumor, with less than 30 cases reported in adults^25^. The same accounts for proximal type epithelioid sarcoma in the mediastinum with even fever reported cases^26–28^. For a better differentiation of these two entities, molecular work-ups might be helpful. Unlike MRT, epithelioid sarcomas harbor in addition to *SMARCB1* (INI1) alterations multiple copy number gains and losses throughout the genome as seen for example in case 1 and 7, in contrast to case 4^19,29^. Other interesting work has shown that epithelioid sarcoma and MRT show differences in miRNA expression, however this might be more challenging to include in a routine clinical work up^30^. In general, pathologists have to be aware of these entities, as two of the cases (patient 4 and 7) were misclassified as mesothelioma due to the tumor location, despite negative common mesothelial markers. Few mesothelioma cases were reported harboring loss of INI1 (SMARCB1) protein expression, but these retained positivity for common mesothelial markers^31,32^.

Principal component analysis of methylation data showed clustering of single patients. Two patients with multiple genomic rearrangements/ complex molecular profiles and a smoking signature (patients 6 and 8) clustered separately. Patient 6 has an intrapulmonary neoplasm with a pattern of metastasis typical for lung carcinoma (lymph nodes, adrenal gland, and liver). Additionally, genomic profile includes mutations in *TP53* and *RB1*, as commonly seen in SCLC and LCNEC. Immunohistochemical co-expression of pan-cytokeratin and claudin4 further support an epithelial lineage. Therefore, this case probably fits best in the category of large cell lung carcinoma with an additional *SMARCB1* alteration as a later event in the evolution of this tumor. Large cell carcinomas are an understudied entity but seem to be closely related to LCNEC and SCLC on a genomic level^31–34^. In the same category falls case 8 with an intrapulmonary lesion with co-expression of pan-cytokeratin and claudin4 and additionally alterations in *TP53* and *RB1*. Classical neuroendocrine morphology and positive synaptophysin immunohistochemistry further support the diagnosis of an LCNEC.

A borderline case is patient 9 with a mass in the posterior mediastinum around the esophagus. This case showed a mixed type histomorphology with tumor cells positive for pan-cytokeratin and patchy for claudin 4. In addition, the molecular profile was more complex with additional alterations in *TP53, MDM2,* and *PARK2* challenging the diagnosis of an epithelioid sarcoma versus a carcinoma. However, the closest methylation class in this case was an epithelioid sarcoma.

Based on our analyses performed, we show that *SMARCB1* (INI1)-deficient neoplasms are very rare and most likely represent a spectrum of known tumor types, namely epithelioid sarcoma, MRT and undifferentiated carcinoma rather than a distinct entity. We elaborate that molecular analyses can help to better categorize these tumors.

A possible limitation of this study is that cases with INI1 (*SMARCB1*) proficient protein expression but potential *SMARCB1* alterations (e.g., mutations) would not have been detected, as retained nuclear staining was a criterion of exclusion in this study. A possible mechanism of loss of nuclear labeling of INI1 (*SMARB1*) immunohistochemistry can be mediated by structural variants involving the not covered intronic regions or through epigenetic or post-translational regulation^35^. However, in the present study and in earlier work no methylation events were detected in the CpGs of the promotor region^18^.

The fact that genes encoding SWI/SNF components are mutated in cancer and show a dismal prognosis raises several key questions, including whether such mutations, despite promoting cancer growth, result in synthetic lethal dependencies. From a therapeutic standpoint, it is of major importance to define whether any such dependencies are specific to the particular subunit that is mutated and/or the tissue of origin, or whether the mutations confer shared synthetic lethal dependencies regardless of which subunit is mutated. Emerging data indicate that mutations in SWI/SNF genes do indeed result in vulnerabilities in cancers, some of which are subunit and/or cell-type specific, although others are potentially more broadly applicable. The pursuit of therapeutic translation is underway for several of these vulnerabilities, with a number of treatment approaches being tested in clinical trials. In tumors with loss of INI1 *(SMARCB1)* clinical and preclinical evidence suggests possible sensitivity to targeted therapies, including EZH2 inhibitors and anti-PD-1 immune checkpoint inhibition^36–39^. Additional new options are on the way, as most recent in vitro data suggest synergistic action of WDR5 and HDM2 inhibitors in *SMARCB1*-deficient cancer cells^40^. We currently face an absence of approved therapies for SMARB1/INI1 deficient tumors but as clinical trials are evolving that include INI1 (*SMARCB1*) deficient tumors it is important to identify these rare individual patients.

We conclude that a proper diagnostic classification of intrathoracic tumors with INI1 (*SMARCB1*) deficiency remains challenging. The diagnostic work up should be guided by histomorphology and immunohistochemistry, without being misguided by tumor location, age or clinical presentation. The differentiation between epithelioid sarcoma, MRT and *SMARCB1* deficient carcinoma might only be possible with the help of molecular profiling. However, an accurate diagnosis is important for best patient care and the correct diagnosis will influence treatment decisions as clinical trials and more targeted therapeutic options are emerging.

## Supplementary information

Supplementary Material

## Data Availability

The datasets used and/or analyzed during the current study are available from the corresponding author on reasonable request.
