# Supplementary material for: Molecular and immunophenotypic characterization of SMARCB1 (INI1) - deficient intrathoracic Neoplasms
Source: Mod Pathol. 2022 Jul 21;35(12):1860–9. doi: 10.1038/s41379-022-01133-4 (PMC9708576; doi:10.1038/s41379-022-01133-4)
Supplement: Supplementary file 1 — Supplementary Material [file 41379_2022_1133_MOESM1_ESM.pdf]

Supplementary Figure 1

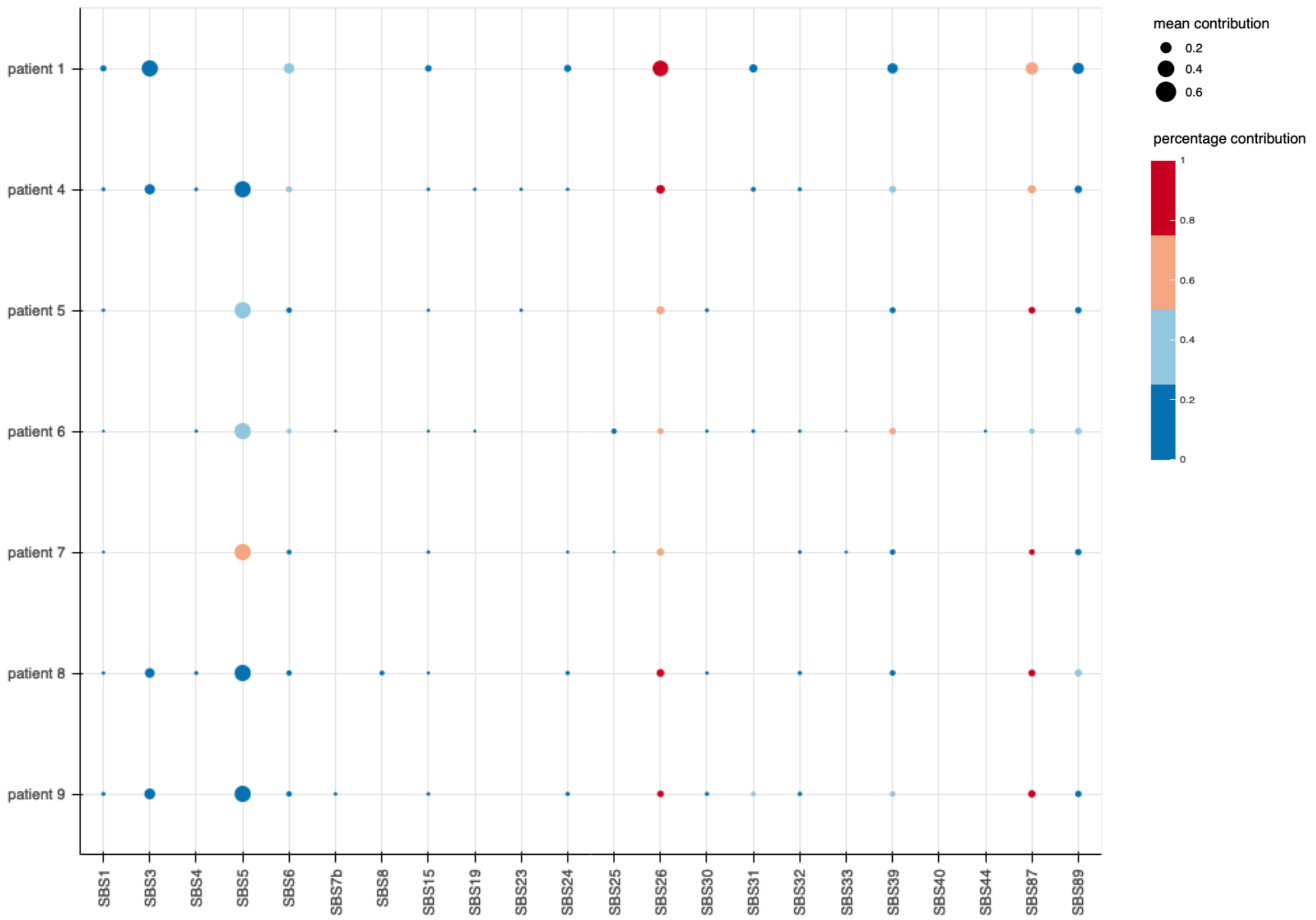

Supplementary Figure 1: COSMIC Mutational Signatures. Dot colors represent the percentage of interactions in which the signature is found, and the size is the average of contributions of that signature after bootstrapping. SBS4-Tobacco smoking only low contribution in only 3 patients (patient 4, 6 and 8). SBS87-Thiopurine chemotherapy treatment shows a high contribution in patients 5, 7, 8 and 9. SBS26-Defective mismatch repair is very stable in patients 1, 4, 8 and 9. SBS5-Unknown (clock-like signature) shows high instability in patients 4, 5, 6, 7, 8, and 9 and high contribution in patient 7.

Supplementary Figure 2

Patient 2:

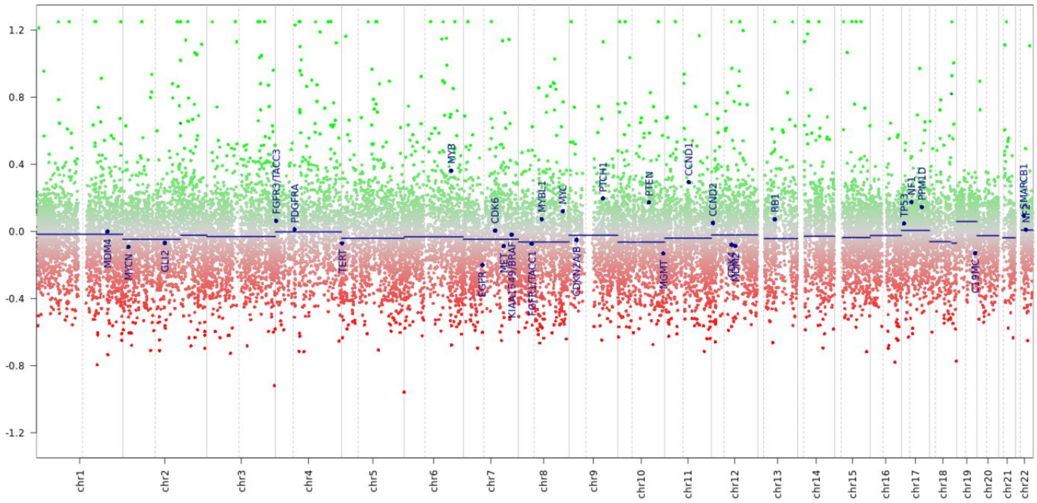

Patient 4:

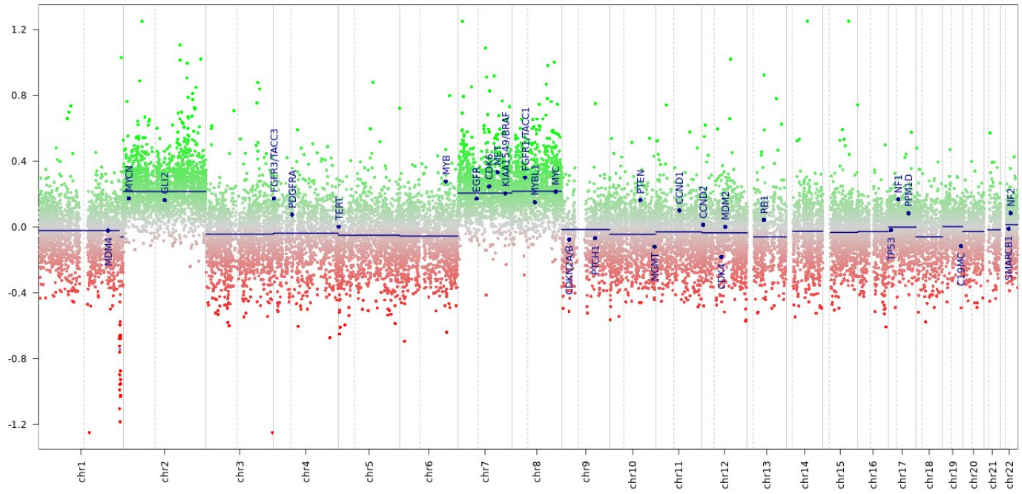

Patient 5:

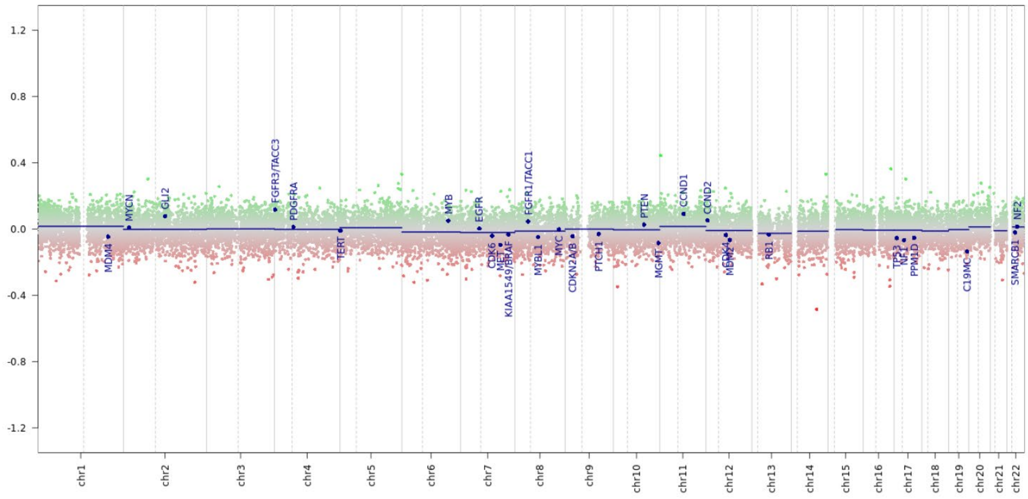

Patient 7:

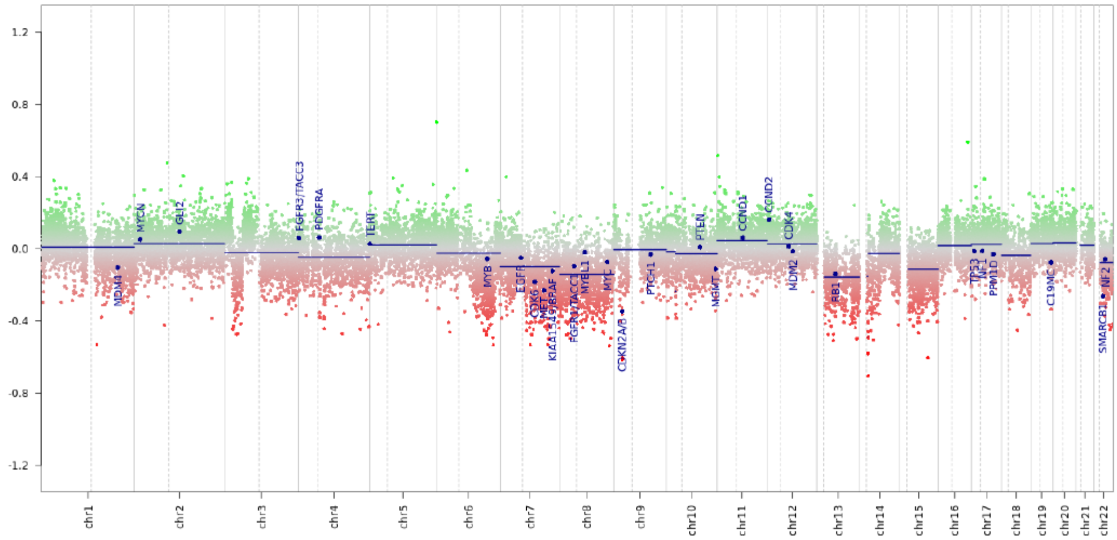

Patient 9:

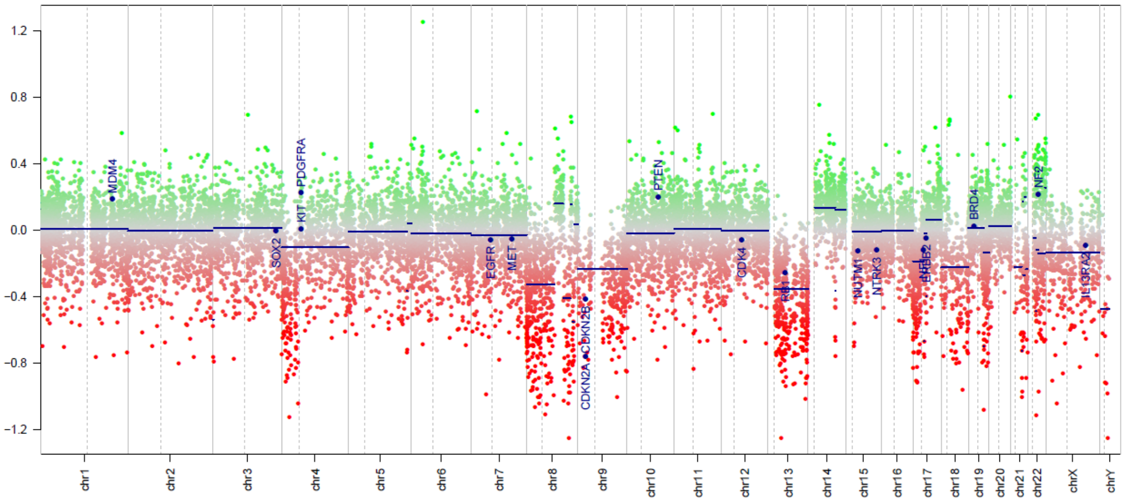

Supplementary Figure 2: Copy number variation (CNV) plots, generated using the Epigenomic Digital Pathology (EpiDiP) platform (www.epidip.org). Patient 1, 3, 6 and 8 were excluded due to poor quality of the plots.

Supplementary Table 1: Retrospective INI1 (*SMARCB1*) - screening of intrathoracic neoplasms

**Histology**	**Total n**	**INI1 loss n (%)**
NSCLC, adenocarcinoma	521	0
NSCLC, squamous cell CA	431	0
NSCLC, mixed type[1]	34	0
Large cell carcinoma	25	1 (4)
SCLC	74	0
LCNEC	60	1 (1.67)
Atypical carcinoid	55	0
Typical carcinoid	83	0
AD-NE mixed	9	0
Pleura mesothelioma	187	1 (0.53)
IHC Screening	1479	3 (0.21)
Sarcoma data base		2
Index cases		4
**Total cases**		**9**

1: adeno-squamous carcinoma. Abbreviations: NSCLC: non-small cell lung cancer, CA: Carcinoma, SCLC: small cell lung carcinoma, LCNEC: large cell neuroendocrine carcinoma, AD-NE mixed: adenocarcinoma mixed with neuroendocrine tumour.

Supplementary Table 2: Antibody, immunohistochemistry protocol information and scoring criteria

Antibody	Clone	Manufacturer	Platform	Antigen Retrieval (min)	Primary antibody incubation	Detection	Dilution	Scoring Criteria
**SMARCB1 (INI1)**	25/BAF47	BD Biosciences	Ventana	CC1 (48)	32 min	OptiView DAB	1:300	loss of nuclear staining
**SMARCA4 (BRG1)**	EPNCIR111A	Abcam	Bond	H2 (90)	30 min	IHC-Refine 30	1:50	loss of nuclear staining
**CKpan b**	AE1/AE3	Dako A/S	Ventana	CC1 (24)	32 min	OptiView DAB	1:50	membranous and cytoplasmic staining
**Claudin 4**	3E2C1	Invitrogen	Bond	H2 (60)	30 min	IHC-Refine 30	1:200	membranous staining
**SMARCA2 (BRM)**	D9E8B	Cell Signaling Technology	Bond	H2 (30)	30 min	IHC-Refine 30	1:800	loss of nuclear staining
**TTF1**	SP141	Ventana-Roche	Ventana	CC1 (64)	12 min	OptiView DAB	prediluted	nuclear staining
**CD34**	QBEnd/10	Ventana-Roche	Ventana	CC1 (16)	32 min	OptiView DAB	prediluted	membranous stain
**Calretinin**	SP65	Ventana-Roche	Ventana	CC1 (24)	16 min	OptiView DAB	prediluted	nuclear and cytoplasmic staining
**Synaptophysin**	27G12	Novacastra	Bond	H2 (40)_97°	30 min	IHC-Refine 30	1:50	cytoplasmic staining
**CD99**	HO36-1.1	Novacastra	Ventana	CC1 16	32 min	OptiView DAB	1:25	nuclear staining
**p40**	BC28	Zytomed Systems	Ventana	CC1 32	32 min	OptiView DAB	1:100	cytoplasmic staining
**WT1**	WT49	Novacastra	Bond	H2(40)	30 min	ICH-Refine 30	1: 40	nuclear staining
**D2-40**	D2-40	Dako A/S	Ventana	CC1 80	32 min	OptiView DAB	1:50	cytoplasmic staining
**CK5/6**	D5/16B4	Ventana-Roche	Ventana	CC1 48	32 min	OptiView DAB	prediluted	cytoplasmic staining

Abbreviation: CC1= EDTA buffer with pH8.4, CC2= Citrat buffer with pH6.0, H2 = EDTA buffer with pH9.0.

Supplementary Table 3: Additional IHC markers, according to the methylation profile and/ or inital diagnosis

**Patient ID**	**1**		**2**		**3**		**4**		**5**		**6**		**7**		**8**		**9**	
**Initial diagnosis**	Thymic carcinoma		Epithelioid sarcoma		SWI/SNF - deficient Neoplasm		Mesothlioma		Malignant epithelioid tumor, favoring ES		Large cell carcinoma of the lung		Mesothlioma		LCNEC		Epithelioid sarcoma	
**Additional marker**	p63	neg.			p40	neg.	WT1	neg.			PAS	neg.	WT1	neg.	Chromo.	neg.		
	p40	neg.					D2-40	neg.			p40	neg.	D2-40	neg.				
	CD5	neg.					CK5/6	neg.					CK5/6	neg.				
							bap1	no loss					bap1	no loss				
							MTAP	no loss					MTAP	no loss				

**Methylation profile (closest match)**	Epithelioid sarcoma		Langerhans cell histiocytosis		failed		malignant rhabdoid tumor (MRT)		Chordoma (dediff.)		Not to classify		Epithelioid sarcoma		Family plexus tumor		Epithelioid sarcoma	
**Additional marker**	ERG	pos.	Langerin	neg.			CD99	neg.	s100	neg					mib-1	>80%		
			CD1	neg.			MyoD	neg.	brachyury	neg.								

Abbreviations: ES: epitheloid sarcoma, LCNEC: Large cell neuroendocrine carcinoma, neg: negative, pso.: positve, Chromo.: Chromogranin.

Supplementary Table 4: Molecular results in SMARCB1-deficient neoplasm

ID	VARIANT-TYPE	GENE	SOMATIC STATUS -FUNCTIONAL IMPACT	SV-PROTEIN-CHANGE	SV-CDS-CHANGE	SV-GENOME-POSITION	SV-COVERAGE	SV-PERCENT-READS	CNA-COPY-NUMBER	CNA-EXONS	CNA-RATIO	CNA-TYPE	BIOMARKER-VALUE
**Patient 1**	**Tumor content: 35%**												
	Tumor Mutational Burden												1.36 Muts/Mb
	Microsatellite Status												MS-Stable
	Loss Of Heterozygosity												1.32%
	copy-number-alteration	SMARCB1	known			chr22:24129150-24176703 (q11.23)			0	10 of 1	0.66	loss	
	copy-number-alteration	CDKN2A	known			chr9:21967751-21995300 (p21.3)			0	5 of 5	0.68	loss	
	short-variant	SOX9	unknown	P266L	797C>T	chr17:70119795	1011	50.74					
	short-variant	POLD1	unknown	T441M	1322C>T	chr19:50909518	852	48.83					
	short-variant	PTCH1	unknown	Y1316C	3947A>G	chr9:98209591	980	49.29					
	short-variant	INPP4B	unknown	V594A	1781T>C	chr4:143045853	1028	53.89					
	short-variant	FOXL2	unknown	P327L	980C>T	chr3:138664585	273	51.28					
	short-variant	EP300	unknown	P1971S	5911C>T	chr22:41573626	1307	50.34					
	short-variant	DDR1	unknown	P564A	1690C>G	chr6:30864574	1079	51.81					
	short-variant	BRIP1	unknown	R419W	1255C>T	chr17:59876546	1161	48.58					
	short-variant	FAM123B	unknown	H134P	401A>C	chrX:63412766	1101	39.87					
	short-variant	FAM123B	unknown	G327A	980G>C	chrX:63412187	1178	21.05					
**Patient 2**	**Tumor content: 45%**												
	Tumor Mutational Burden												0.88 Muts/Mb
	Microsatellite Status												MS-Stable
	Loss Of Heterozygosity												1.18%
	short-variant	PARK2	known	T240M	719C>T	chr6:162394349	771	51.62					
	short-variant	MYC	unknown	S362F	1085C>T	chr8:128752924	1626	49.69					
	short-variant	PIK3C2B	unknown	R458Q	1373G>A	chr1:204429727	807	47.83					
	short-variant	MKNK2	unknown	D32N	94G>A	chr19:2046648	871	49.94					
	short-variant	MLL	unknown	R185Q	554G>A	chr11:118342428	929	48.65					
	short-variant	HGF	unknown	R373G	1117C>G	chr7:81355257	918	23.97					
	short-variant	GATA2	unknown	P161A	481C>G	chr3:128204960	943	50.69					
	short-variant	FAT1	unknown	S3581G	10741A>G	chr4:187524939	1413	49.04					
**Patient 3**	**Tumor content: 80%**												
	Tumor Mutational Burden												3.26 Muts/Mb
	Microsatellite Status												MS-Stable
	Loss Of Heterozygosity												0.1 %
	short-variant	ATM	known	R2227C	6679C>T	chr11:108196143	317	55.52					
	short-variant	SMARCB1	likely	T250fs*17	747_748CA>G	chr22:24159075	291	71.48					
	short-variant	APCDD1	unknown	K454E	1360A>G	chr18:10487850	248	46.37					
	short-variant	AXIN1	unknown	A745V	2234C>T	chr16:341250	165	53.94					
	short-variant	BRCA2	unknown	K3326*	9976A>T	chr13:32972626	443	44.92					
	short-variant	MLL2	unknown	N2944S	8831A>G	chr12:49432308	363	49.31					
	short-variant	PIK3C2G	unknown	R981L	2942G>T	chr12:18656263	272	47.79					
	short-variant	PIM1	unknown	E124Q	370G>C	chr6:37139030	355	46.76					
	short-variant	SOX10	unknown	G267R	799G>A	chr22:38370104	270	38.52					
**Patient 4**	**Tumor content: 85%**												
	Tumor Mutational Burden												4.07 Muts/Mb
	Microsatellite Status												MS-Stable
	Loss Of Heterozygosity												0.1 %
	copy-number-alteration	SMARCB1	known			chr22:24129150-24176703 (q11.23)			0	1 of 10	.12	loss	
	short-variant	ASMTL	unknown	R79W	235C>T	chrX:1558028	630	48.10					
	short-variant	BARD1	unknown	S310A	928T>G	chr2:215645670	1031	34.04					
	short-variant	EPHA5	unknown	G783A	2348G>C	chr4:66217267	604	46.69					
	short-variant	FLCN	unknown	R320Q	959G>A	chr17:17122436	687	48.18					
	short-variant	MRE11A	unknown	N556S	1667A>G	chr11:94180501	671	47.99					
	short-variant	TSC2	unknown	H270R	809A>G	chr16:2107140	429	39.16					
**Patient 5**	**Tumor content: 50%**												
	Tumor Mutational Burden												1.63 Muts/Mb
	Microsatellite Status												MS-Stable
	Loss Of Heterozygosity												0.10%
	short-variant	MUTYH	known	Y165C	494A>G	chr1:45798475	764	46.73					
	short-variant	SMARCB1	likely	E246*	736G>T	chr22:24159064	631	20.92					
	short-variant	SMARCB1	likely	K296fs*24	888delG	chr22:24167503	835	18.80					
	short-variant	AXL	unknown	Q361P	1082A>C	chr19:41744462	548	46.53					
	short-variant	CHEK2	unknown	T378I	1133C>T	chr22:29091824	498	42.17					
	short-variant	EPHA7	unknown	N361K	1083C>G	chr6:94066676	897	51.28					
	short-variant	EXOSC6	unknown	L122M	364C>A	chr16:70285440	76	51.32					
	short-variant	FANCA	unknown	E1293K	3877G>A	chr16:89806459	621	47.99					
	short-variant	FAT3	unknown	T1940S	5818A>T	chr11:92531997	785	23.31					
	short-variant	MAP3K6	unknown	K1125del	3373_3375delAAG	chr1:27683229	457	50.33					
	short-variant	PIK3C3	unknown	T126S	376A>T	chr18:39542572	688	48.84					
**Patient 6**	**Tumor content: 60%**												
	Tumor Mutational Burden												6.52 Muts/Mb
	Microsatellite Status												MS-Stable
	Loss Of Heterozygosity												26.5 %
	short-variant	NFE2L2	known	L30F	88C>T	chr2:178098957	913	64.40					
	short-variant	RB1	known	R579*	1734_1735CC>TT	chr13:49027167	628	42.83					
	short-variant	TP53	known	V157F	469G>T	chr17:7578461	487	60.57					
	copy-number-alteration	FGF10	known						9	3 of 3	2.11	amplification	
	copy-number-alteration	NKX2-1	known						25	5 of 5	5.62	amplification	
	copy-number-alteration	RAD21	known						8	12 of 1	2.28	amplification	
	copy-number-alteration	RICTOR	known						8	33 of 3	2.36	amplification	
	copy-number-alteration	SMARCB1	known						0	5 of 10	.36	loss	
	short-variant	FLCN	likely	Q533*	1597C>T	chr17:17117112	545	81.65					
	short-variant	CASP8	unknown	M1?	-82_35>G	chr2:202122873	702	11.82					
	short-variant	CBFB	unknown	L184F	550C>T	chr16:67132667	713	63.53					
	short-variant	FLT4	unknown	A786D	2357C>A	chr5:180047658	335	59.70					
	short-variant	HNF1A	unknown	Q170H	510G>C	chr12:121426819	398	13.82					
	short-variant	MAP3K1	unknown	R248Q	743G>A	chr5:56155651	846	17.61					
	short-variant	MED12	unknown	R1735H	5204G>A	chrX:70356309	381	8.14					
	short-variant	MED12	unknown	I320M	960A>G	chrX:70341525	497	68.01					
	short-variant	NCOR2	unknown	D377G	1130A>G	chr12:124914178	330	42.12					
	short-variant	PAG1	unknown	V230M	688G>A	chr8:81897199	1415	17.17					
	short-variant	SPEN	unknown	G112A	335G>C	chr1:16199562	834	15.71					
	short-variant	TMEM30A	unknown	V234E	701T>A	chr6:75968687	771	62.13					
	short-variant	WT1	unknown	G60R	178G>A	chr11:32456714	115	83.48					
	copy-number-alteration	APCDD1	unknown						7	5 of 5	1.67	amplification	
	copy-number-alteration	IL7R	unknown						8	8 of 8	1.78	amplification	
	copy-number-alteration	MIB1	unknown						7	21 of 2	2.22	amplification	
	copy-number-alteration	NBN	unknown						8	14 of 1	2.33	amplification	
	copy-number-alteration	PTPN2	unknown						7	12 of 1	2.27	amplification	
**Patient 7**	**Tumor content: 60%**												
	Tumor Mutational Burden												3.26 Muts/Mb
	Microsatellite Status												MS-Stable
	Loss Of Heterozygosity												8.3 %
	short-variant	ATM	known	E2039K	6115G>A	chr11:108186757	616	1.46					
	copy-number-alteration	CDKN2A	known						0	5 of 5	.70	loss	
	copy-number-alteration	CDKN2B	known						0	5 of 5	.71	loss	
	short-variant	DNMT3A	likely	R598*	1792C>T	chr2:25467083	378	7.94					
	short-variant	CD22	unknown	N771H	2311A>C	chr19:35836607	469	50.75					
	short-variant	DTX1	unknown	V613I	1837G>A	chr12:113534718	251	20.32					
	short-variant	FAM46C	unknown	S5N	14G>A	chr1:118165504	318	39.62					
	short-variant	FLT1	unknown	S733del	2178_2180delATC	chr13:28942736	175	55.43					
	short-variant	IKBKE	unknown	I370M	1110C>G	chr1:206652403	432	43.52					
	short-variant	JAK2	unknown	C384R	1150T>C	chr9:5064976	648	50.31					
	short-variant	MSH6	unknown	D803G	2408A>G	chr2:48027530	640	49.53					
	short-variant	MYO18A	unknown	R555H	1664G>A	chr17:27447698	476	49.79					
	short-variant	RPL11	unknown	splice site	6+1G>T	chr1:24018320	417	17.03					
	short-variant	SMARCA1	unknown	splice site	1662+2T>C	chrX:128627015	257	40.08					
	short-variant	SMARCA4	unknown	R1618Q	4853G>A	chr19:11170805	273	47.25					
	short-variant	TRAF5	unknown	E262fs*10	786delA	chr1:211538806	433	55.89					
	short-variant	TRAF5	unknown	E219D	657G>T	chr1:211534465	451	51.88					

**Patient 8**	**Tumor content: 60%**												
	Tumor Mutational Burden												6.52 Muts/Mb
	Microsatellite Status												MS-Stable
	Loss Of Heterozygosity												8.5 %
	copy-number-alteration	FGF12	ambiguous						8	6 of 6	2.46	amplification	
	copy-number-alteration	PIK3CA	ambiguous						8	20 of 2	2.35	amplification	
	copy-number-alteration	SOX2	ambiguous						8	5 of 5	2.23	amplification	
	copy-number-alteration	TBL1XR1	ambiguous						8	14 of 1	2.41	amplification	
	short-variant	CKS1B	known	E18Q	52G>C	chr1:154947273	958	1.04					
	short-variant	TP53	known	Y236C	707A>G	chr17:7577574	549	83.24					
	copy-number-alteration	FRS2	known						24	5 of 5	6.06	amplification	
	copy-number-alteration	MDM2	known						20	11 of 1	5.66	amplification	
	copy-number-alteration	SMARCB1	known						0	2 of 10	.13	loss	
	short-variant	RB1	likely	I214fs*4	639_642delGATT	chr13:48934183	399	69.17					
	short-variant	CPS1	unknown	M1312I	3936G>T	chr2:211527855	478	5.65					
	short-variant	ESR1	unknown	S118P	352T>C	chr6:152129399	315	47.62					
	short-variant	FAT3	unknown	R218Q	653G>A	chr11:92085931	815	60.49					
	short-variant	KIT	unknown	K778E	2332A>G	chr4:55598135	597	37.69					
	short-variant	LRP1B	unknown	S1835N	5504G>A	chr2:141528572	674	62.17					
	short-variant	LRP1B	unknown	R2219C	6655C>T	chr2:141457963	979	63.02					
	short-variant	MSH6	unknown	E220D	660A>C	chr2:48025782	659	30.65					
	short-variant	PIK3C3	unknown	E150*	448G>T	chr18:39550337	1046	42.35					
	short-variant	PTEN	unknown	R159W	475A>T	chr10:89692991	560	76.43					
	short-variant	RAD50	unknown	E18Q	52G>C	chr5:131893068	665	15.79					
	short-variant	RELN	unknown	T1873I	5618C>T	chr7:103197603	670	63.28					
	copy-number-alteration	ATR	unknown						8	47 of 4	2.33	amplification	
	copy-number-alteration	BCL6	unknown						8	8 of 8	2.08	amplification	
	copy-number-alteration	KLHL6	unknown						8	7 of 7	2.31	amplification	
	copy-number-alteration	MAP3K13	unknown						8	15 of 1	2.31	amplification	
	copy-number-alteration	RPL35A	unknown						8	4 of 4	2.13	amplification	
	copy-number-alteration	TIPARP	unknown						8	5 of 5	2.25	amplification	
	copy-number-alteration	TP63	unknown						8	16 of 1	2.81	amplification	
**Patient 9**	**Tumor content: 80%**												
	Tumor Mutational Burden												5.7 Muts/Mb
	Microsatellite Status												MS-Stable
	Loss Of Heterozygosity												0.05
	short-variant	TP53	known	R282W	844C>T	chr17:7577094	643	45.57					
	copy-number-alteration	MAPK1	known						11	8 of 8	4.11	amplification	
	copy-number-alteration	SMARCB1	known						0	6 of 10	.38	loss	
	short-variant	NOTCH1	likely	Y550fs*1	1649_1650insA	chr9:139410452	422	7.82					
	short-variant	ARID1A	unknown	E1779G	5336A>G	chr1:27105725	560	73.04					
	short-variant	ASXL1	unknown	A1022G	3065C>G	chr20:31023580	757	47.56					
	short-variant	BCL11B	unknown	T131M	392C>T	chr14:99723843	803	36.86					
	short-variant	GRIN2A	unknown	T1064A	3190A>G	chr16:9858211	733	45.16					
	short-variant	IRF8	unknown	R170G	508C>G	chr16:85946797	682	48.09					
	short-variant	MYO18A	unknown	S314R	942C>G	chr17:27493017	639	50.70					
	short-variant	NTRK1	unknown	G18E	53G>A	chr1:156830779	413	49.15					
	short-variant	NTRK3	unknown	L115P	344T>C	chr15:88726700	692	29.19					
	short-variant	PIK3R1	unknown	D168E	504T>A	chr5:67575431	497	51.71					
	short-variant	RAD50	unknown	V127I	379G>A	chr5:131915022	554	45.31					
	short-variant	RAF1	unknown	R41Q	122G>A	chr3:12660099	728	48.49					
	short-variant	SOX2	unknown	G13A	38G>C	chr3:181430186	185	43.78					
	short-variant	TLL2	unknown	R711C	2131C>T	chr10:98144407	729	32.92					
	short-variant	WDR90	unknown	R1290C	3868C>T	chr16:711894	600	30.83					
